# Estradiol reduction through aromatase inhibition impairs cocaine seeking in male rats

**DOI:** 10.3389/fnbeh.2023.1307606

**Published:** 2024-01-16

**Authors:** John K. Alvarado-Torres, Roberto Morales-Silva, Alexia Sanabria Ponce de Leon, Genesis Rodriguez-Torres, Joshua Perez-Torres, Yobet Perez-Perez, Devin Mueller, Marian T. Sepulveda-Orengo

**Affiliations:** ^1^Basic Sciences Department, Ponce Research Institute, Ponce Health Sciences University, Ponce, Puerto Rico; ^2^Department of Biomedical Sciences, Pontifical Catholic University of Puerto Rico, Ponce, Puerto Rico; ^3^Department of Biological Sciences, Kent State University, Kent, OH, United States

**Keywords:** cocaine, estradiol, conditioned place preference, extinction, self-administration, rats, aromatase inhibitor

## Abstract

**Introduction:**

Clinical and preclinical research on cocaine use disorder (CUD) has shown that sex differences in drug seeking are influenced by hormonal fluctuations. Estradiol (E2), a sex steroid hormone, has been linked to female drug effects, vulnerability to use/abuse, and psychosocial factors. Preclinical studies show that estradiol in females facilitates the extinction of cocaine-seeking behavior indicating a possible role in regulating extinction learning. Similar to females, males’ brains contain the aromatase enzyme which converts testosterone to estradiol. However, it is unclear whether estradiol plays a role in male extinction learning as it does in females. Furthermore, how endogenously aromatized estradiol affects drug addiction in males is unknown. Therefore, this study investigated whether endogenous estradiol regulates cocaine seeking in male rats. We hypothesized that decreased aromatase enzyme activity, resulting in decreased estradiol synthesis in male brains, will impair extinction learning leading to increased cocaine-seeking behavior.

**Methods:**

This hypothesis was tested using cocaine-conditioned place preference (CPP), and short access self-administration (SA), followed by extinction and reinstatement. Before each extinction session for CPP or SA, male rats received an injection of either 1 (low dose) or 2.5 mg/kg (high dose) of the aromatase inhibitor Fadrozole (FAD), or vehicle.

**Results:**

FAD groups showed dose-dependent effects on cocaine-seeking behavior compared to the vehicle group during CPP extinction. Specifically, low dose FAD facilitated extinction of cocaine CPP, whereas high dose FAD impaired it. In contrast, neither dose of FAD had any effects on the extinction of cocaine SA. Interestingly, only the low dose FAD group had decreased active lever pressing during cue- and cocaine-primed reinstatement compared to the vehicle group. Neither dose of FAD had an effect on sucrose extinction or reinstatement of sucrose seeking.

**Discussion:**

These results from CPP experiments suggest that estradiol may impact extinction learning, as a low dose of FAD may strengthen the formation of cocaine extinction memory. Additionally, in male rats undergoing cocaine SA, the same low dose of aromatase inhibitor effectively reduced reinstatement of cocaine-seeking behavior. Thus, estradiol impacts cocaine seeking and extinction in both males and females, and it may also influence the development of sex-specific treatment strategies for CUD.

## 1 Introduction

Sexual dimorphism in drug addiction has been extensively studied over the last three decades (Robbins et al., 1999; Fattore et al., 2008; Becker et al., 2017; Peterson et al., 2020; Torres, 2022) focusing on hormonal variances and the possibility of treatments tailored to sex differences. Clinical studies focused on the neurobiology of addiction have shown that women are at higher risk of addictive disorders than men (Jackson et al., 2006; Anker and Carroll, 2011; Kerstetter and Kippin, 2011). More significantly, estrogens, which have been studied from a sex-difference perspective in multiple drug-addictive disorders in which differences between males and females are observable (Rubonis et al., 1994; Dudish and Hatsukami, 1996; Holly et al., 2012), is viewed as one of the primary causes of sex differences (Hu et al., 2004; Jackson et al., 2006; Lynch, 2008). Interestingly, estrogens are also synthesized *de novo* in the brain (Hojo et al., 2004) where they are associated with neuroprotection (Samantaray et al., 2016) and are necessary for synaptic plasticity (Hojo et al., 2008), learning, and memory (Rosenfeld et al., 2018). Within the male and female physiological system, estrogens are required for brain development (Wu et al., 2009) and neuroprotection (Zendedel et al., 2018; Wang et al., 2021).

Up until the discovery that sex differences contribute to a wide range of diseases (Schuurs and Verheul, 1990; Scheidt-Nave et al., 1991; Jacobs et al., 1992), research was conducted almost exclusively in males for many decades (Mastroianni et al., 1994). As the relevance of conducting research on both males and females became recognized (Wald and Wu, 2010), new studies revealed how in many instances hormones produced sex-dependent differences, including in addictive disorders (Robbins et al., 1999; Fattore et al., 2008; Becker et al., 2017; Peterson et al., 2020). In cocaine use disorder, susceptibility of females (but not of males) can be altered by estradiol (E2), which heightens the responsiveness of the brain’s reward system (Jackson et al., 2006; Galankin et al., 2010; Martinez et al., 2016). Additionally, female rats exposed to cocaine self-administration (SA) learn to seek out and consume the drug faster than their male counterparts (Lynch, 2008). Despite an increased understanding of E2 effects in females, whether E2 was important for drug seeking in males has been neglected.

There are several types of estrogens, with estradiol being the most potent form. E2 is known to modulate cocaine addiction in females by increasing the sensitivity of the brain reward system (Galankin et al., 2010; Martinez et al., 2016). In a cocaine SA paradigm, female rats acquire cocaine-seeking behavior more readily than males (Lynch, 2008). Similarly, in a cocaine-conditioned place preference (CPP) paradigm where female rats were grouped by proestrus/oestrous cycles (when the levels of estradiol are higher) and dioestrus I and II (when cycles the levels of estrogen are lower) cycles, the proestrus/oestrus group showed enhanced cue/reward association (Zysow et al., 1997; Goldman et al., 2007; Becker and Hu, 2008; Calipari et al., 2017). Importantly, Twining et al. (2013) found that E2 not only enhances the magnitude of a CPP but is also necessary for extinction in female rats. In particular, ovariectomized female rats showed persistent CPP that did not extinguish across more than forty tests, but injecting E2 rescued extinction (Twining et al., 2013). Thus, E2 plays a central role in cocaine seeking and extinction in females. In males, however, little is known about the role of E2 in cocaine seeking and extinction. The impact of E2 on the cocaine-reinforcing effects in males is still unclear. One study found that E2 administration increased cocaine SA in female rats but had no effect on gonadectomized male rats (Jackson et al., 2006). However, another study found that chronic administration of E2 in male rats increased cocaine choice over food during concurrent reinforcement and under progressive ratio (Bagley et al., 2019). These studies investigated the impact of E2 on the reinforcing effects of cocaine, and we extend these findings to include extinction and reinstatement.

Relevant to our study, Graham and Milad (2014) have shown that inhibiting the production of E2 by the aromatase inhibitor, Fadrozole (FAD), negatively affects the ability of male rats to extinguish conditioned fear. The results of this study suggest that E2 is required for the successful extinction of learned fear, prompting us to investigate the role of E2 in the extinction of drug seeking in males. Therefore, we hypothesize that E2 is a mediator of extinction of cocaine seeking in both females and males.

Aromatization of androgens to estrogens along the cholesterol pathway is the last step in steroid synthesis. The aromatase enzyme, which is a Cytochrome P450 protein found in many body parts (the brain, liver, breast, and fat; Rochira et al., 2000; Stocco, 2012), and the aromatase inhibitors that are currently available, are valuable tools for the study of male brain estrogens and their potential role in substance use disorders. FAD, a potent non-steroidal second-generation aromatase inhibitor, can efficiently halt brain synthesis of E2 (Afonso et al., 2000).

The aim of the present study is to examine if endogenous E2 is involved in the extinction of cocaine seeking. Two behavioral paradigms for cocaine use disorder were used to determine the role of E2 in the extinction of cocaine seeking: CPP and SA. We hypothesized that FAD enhances extinction of cocaine CPP and SA leading to a reduced cocaine-seeking behavior in male rats.

## 2 Materials and methods

### 2.1 Animals

Animals were provided by the local Animal House (Ponce Research Institute). All procedures were approved by the Ponce Health Sciences University Institutional Animal Care and Use Committee (Protocol #2103000487), based on the Guide for the Care and Use of Laboratory Animals published by the National Institutes of Health [NIH; National Research Council (U.S.), Committee for the Update of the Guide for the Care and Use of Laboratory Animals, and Institute for Laboratory Animal Research (U.S.), 2011]. At the beginning of the study, male Long Evans rats (CPP n = 53 and SA n = 52) were selected based on age (~P60) and weight (250 g to 300 g). Rats were on a 12-h light/dark cycle. For cocaine CPP experiments, rats were pair-housed in standard plastic cages (clear) and fed with pellet chow and water *ad libitum*, except when placed in experiment boxes. CPP experiments took place during the light phase. For cocaine SA, rats were single housed in standard clear plastic cages, fed an 18 g/day diet of pellet chow and had water *ad libitum*. SA experiments took place during the dark phase.

### 2.2 Surgeries

#### 2.2.1 Intrajugular catheterization

Rats were anesthetized with Isoflurane (2–4%) in oxygen throughout the procedure. Body heat was maintained throughout the surgical procedure using heating pads. For catheter implantation, a guide cannula (C313G, *Plastics One*) attached to silastic tubing (0.025 ID, 0.047 OD *Bio-sill*) was inserted subcutaneously between the shoulder blades and exited the skin via a dermal biopsy hole (3 mm). Catheters exiting the skin were secured by a subdermal surgical mesh (*Atrium*) and by a cannula. The other end of the catheter was inserted 3 cm into the right jugular vein and securely sutured to the underlying epithelial tissue. A catheter cap was used when the rats were not connected to infusion pumps. Before behavioral protocols, animals were provided seven days to recover from surgery. Catheters were flushed daily with 0.1 mL each of 5 mg/mL gentamicin and 70 U/mL heparinized saline to maintain catheter patency. Catheter testing for patency was performed one day before and after SA with propofol (1 mg/0.1 mL, i.v.).

### 2.3 Drugs

Cocaine hydrochloride (provided by the NIDA drug supply program) was dissolved in sterile saline (0.9% solution) to a concentration of 10 mg/mL or 5 mg/mL. During the conditioning phase of CPP, a cocaine dose of 10 mg/kg was administered intraperitoneally. In SA, cocaine was infused at 0.25 mg/kg/infusion.

Fadrozole hydrochloride *(Sigma Aldrich* F3806-50MG 121M4604V and *Med Chem Cas* no. 102676–31-3 Lot# 42554) was dissolved in sterile saline (0.9% solution) to a concentration of 1.0 and 2.5 mg/mL. During the extinction phase of CPP or SA, FAD was administered intraperitoneally based on group assignments (1.0, or 2.5 mg/kg). Sterile saline was used for control or vehicle solution (0.9% solution).

### 2.4 Behavioral testing

#### 2.4.1 Conditioned place preference

Testing and conditioning were conducted in a 3-chamber apparatus in which two larger conditioning chambers (33 × 23 × 29 cm) were separated by a smaller chamber (15 × 18 × 29 cm). The larger conditioning chambers had wire mesh with white walls in one and gold-grated flooring with a black wall in the other. The center chamber had aluminum sheeting as flooring. All floors were raised 4 cm with removable trays placed beneath. Removable partitions were used to isolate the rats within specific chambers during conditioning. During baseline and CPP trials, the doors were removed to allow free access to the entire apparatus. Each of the larger chambers contained two infrared photo beams separated by 8 cm. If the beam furthest from the door was broken, the rat was considered to be in the larger chamber. If only the beam closest to the center chamber was broken, then the rat was considered to be in the center chamber.

During all phases of the experiments, the room was kept in semi-darkness. A pre-test determined baseline preferences by placing the rats into the center chamber with free access to the entire apparatus for 15 min and recording time in each chamber. Rats spent equal time in the larger conditioning chambers, and less time in the center chamber as previously reported (Yousuf et al., 2019). After the acclimation period, rats began cocaine place conditioning training. All sessions were scheduled between 0700 and 1,900 h. (light-cycle). On alternating days, rats were either injected with cocaine (10 mg/kg, i.p.) before placement in one chamber or with a saline injection in another chamber until they developed an association between the drug and the chamber (Mueller and Stewart, 2000). Two CPP experiments were conducted as follows.

##### 2.4.1.1 CPP experiment 1

Short conditioning of 8 days in total was conducted, with four cocaine and four saline pairings. After injection, rats were confined to the assigned chamber for 20 min each day. After conditioning, rats were assigned to different treatment groups for extinction. Thirty min before extinction training, rats were administered either saline or FAD (1.0 mg/kg). Then, all rats were given free access to the entire apparatus for 15 min daily in the absence of cocaine or saline administration until there was no significant difference in time spent in both cocaine- and saline-paired chambers.

##### 2.4.1.2 CPP experiment 2

Extended conditioning of 12 days was conducted, with six days of cocaine and six days of saline pairings. During conditioning, time in the assigned chamber was 20 min each day. Thirty min before extinction training, rats were administered either saline or one of two FAD doses (1.0, or 2.5 mg/kg). Each extinction sessions lasted 30 min.

#### 2.4.2 Self-Administration

Rats were individually housed and, after the handling period (~5 days), had food removed for 24 h before the food training session in operant conditioning chambers (Med Associates) controlled by computer software (MedPC V). This session allowed association between lever pressing and sucrose pellets. The operant chamber had two levers: an active lever (which dispenses sucrose) and an inactive one (no action). This protocol ran until all rats reached the set criteria (>200 presses), after which rats underwent surgery for intrajugular catheterization (maintenance of the catheter is described in the surgery section above). The day before the onset of SA conditioning (which started after five days of recovery from surgery), all rats were tested for patency using 0.1–0.2 cc of Propofol 1%. For the conditioning phase, rats were placed inside an operant conditioning chamber and connected to an infusion line. Cocaine was self-administered on a fixed-ratio 1 (FR1) schedule of reinforcement (2 h per day). Active lever presses resulted in a drug infusion, a 5 s compound stimulus (cue light and tone, 70 dB) and a 20 s time-out period. Cocaine dose was 5 mg/mL and infused at 0.25 mg/kg/infusion (0.020 mL total volume of infusion per 300 g rat). Criteria for self-administration were ten days of at least ten infusions per session. Rats were divided into treatment groups based on active lever presses during the last three days of SA. Following self-administration of cocaine, rats received extinction training in which the active lever was no longer paired with the light, tone, or an infusion of cocaine for 15 days. Fadrozole (1.0 or 2.5 mg/kg) or vehicle (i.p.) was injected 30 min prior to each extinction session (2 h). After extinction, rats received a cue-primed (tone and light) reinstatement test (2 h), followed by two days of extinction and a subsequent cocaine-primed reinstatement test (2 h, 10 mg/kg, i.p.).

To evaluate the response to non-drug reward, we conducted a separate experiment. Rats were trained to self-administer sucrose (FR1; 45 mg/pellet, *TestDiet*, Richmond, IL) in the same operant conditioning chambers used for cocaine experiments. Experimental conditions remained the same with the same number of days of SA and extinction as cocaine-administering rats, followed by a cue-primed sucrose reinstatement test (2 h). Rats were injected with FAD (1.0 or 2.5 mg/kg) or vehicle (i.p.) 30 min prior to each extinction session, as in cocaine experiments.

### 2.5 Data analysis

Statistical analyses were performed using Graph Pad Prism. CPP and SA behavioral results were assessed using two-way ANOVA repeated-measures analyses. Significant main effects were followed by Bonferroni *post hoc* tests. Values were reported as the mean ± the standard error of the mean (S.E.M.), and significance was considered as *p < 0.05* (*p < 0.05).

## 3 Results

### 3.1 Fadrozole prevents the expression of cocaine seeking in extinction

**Figure 1:**
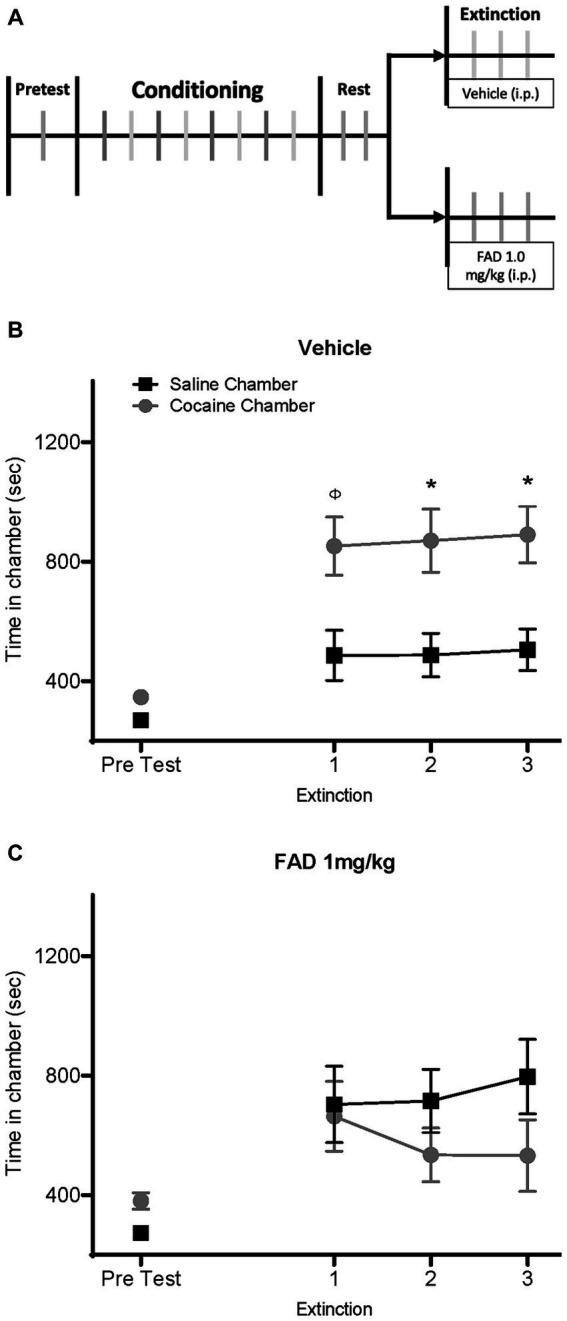
FAD prevents expression of cocaine seeking in extinction. **(A)** Timeline of experiment with male rats receiving an i.p. injection of Fadrozole or vehicle during extinction after cocaine CPP. **(B)** Vehicle-treated rats (*n* = 12) showed a cocaine CPP across all three days of extinction. **(C)** Male rats injected daily with Fadrozole (1 mg/kg, i.p., *n* = 12) during extinction exhibited a blunt expression of drug seeking. *cocaine vs. saline chamber, *p* < 0.05; φ = 0.0545.

To test whether E2 affects the expression and extinction of cocaine seeking in males, we conducted a cocaine CPP experiment using an aromatase inhibitor (FAD) to suppress E2 synthesis in the brain. Male rats were divided into two groups and injected with either saline or FAD (1 mg/kg, i.p.) daily, 30 min prior to each extinction session (Figure 1A). As expected, the saline-treated group exhibited a CPP (Figure 1B). Two-way ANOVA repeated-measures analysis indicated a significant maiF effect of Chamber Time × Sessions, and CPP extinction trials (*F*_8,176_ = 5.727, *p* < 0.0001; *F*_8,176_ = 9.970, *p* < 0.0001; respectively) for the vehicle group. Bonferroni *post hoc* analyses confirmed CPP expression during the second and third extinction trials in the vehicle group, but not in the FAD group (*p* < 0.05; Figure 1B). In the FAD-treated group, however, there was no expression of cocaine seeking as indicated by the lack of CPP (Figure 1C). No significant difference between time in conditioning chambers during extinction sessions were observed (all *ps* > 0.05). These results suggest that suppression of E2 synthesis by inhibiting aromatase activity interferes with cocaine CPP.

### 3.2 Fadrozole induces dose-dependent effects on extinction of cocaine CPP

**Figure 2:**
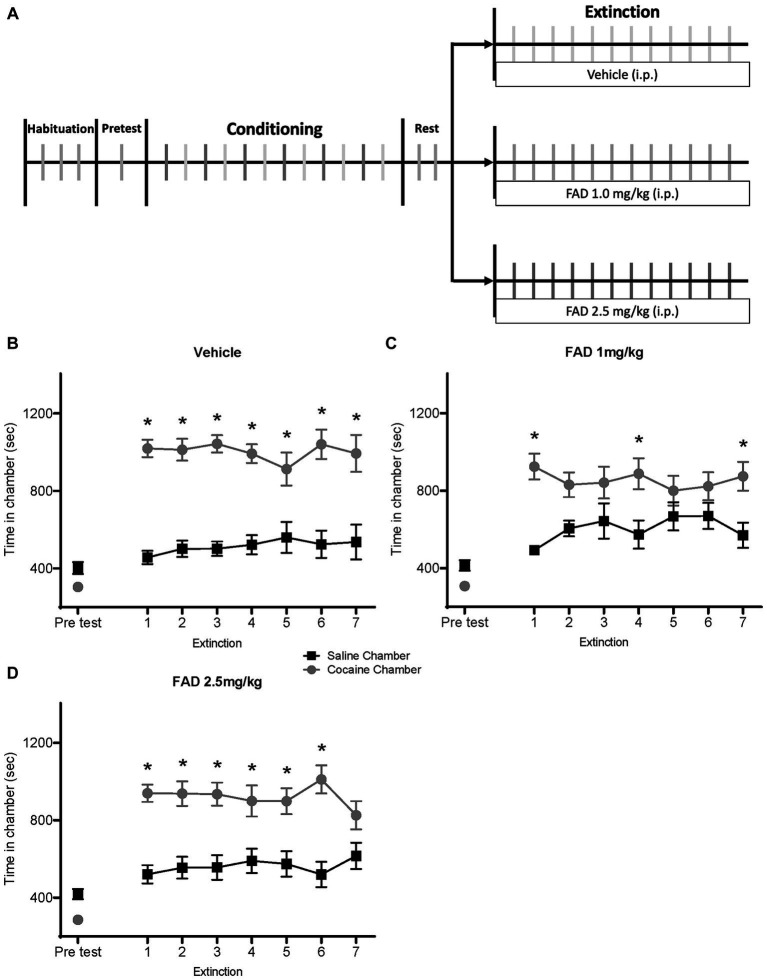
FAD 1.0 mg/kg facilitates whereas 2.5 mg/kg impairs extinction of cocaine-seeking behavior. **(A)** Schematic outline for the experimental timeline of cocaine CPP and i.p. injection of treatment (vehicle, FAD 1.0 mg/kg, FAD 2.5 mg/kg) during extinction. **(B)** Graph shows preference for cocaine or saline chambers starting with an initial pretest of 15 min and followed by extinction sessions of 30 min per day. Vehicle-treated rats (*n* = 13) showed a robust CPP across days followed by extinction. **(C)** Low dose (1 mg/kg) FAD-treated rats (*n* = 15) showed CPP on day one and rapid extinction. **(D)** High dose (2.5 mg/kg) FAD-treated rats (*n* = 16) showed a robust CPP that was resistant to extinction. *cocaine vs. saline chamber, *p* < 0.05.

We examined different doses of FAD during extinction of cocaine seeking. Using the CPP paradigm, rats were conditioned with cocaine and then given extinction training (Figure 2A). Rats were divided into three groups (vehicle, FAD 1.0 mg/kg, and FAD 2.5 mg/kg), and injections were administered daily 30 min. Prior to each extinction session.

The vehicle-treated group showed a CPP for the cocaine-paired chamber across days (Figure 2B). ANOVA revealed differences between time spent in the cocaine- and the saline-paired chamber across extinction trials for the vehicle group (Figure 2B; Chamber Time x Sessions *F*_7,168_ = 10.91, *p* < 0.0001). The FAD 1.0 mg/kg treatment group expressed cocaine CPP on the first day, and showed rapid extinction (Figure 2C; Chamber Time × Sessions *F*_7,196_ = 4.359, *p* = 0.0002). *Post hoc* analyses confirmed that low doses of FAD altered CPP expression on some extinction days, except days one, four, and seven, whereas high-dose FAD impaired extinction and maintained a persistent CPP. The group that received FAD 2.5 mg/kg showed impaired extinction compared to the vehicle group, maintaining a constant CPP across multiple days (Figure 2D; Chamber Time × Sessions *F*_7, 210_ = 9.477, *p* < 0.0001).

### 3.3 Aromatase inhibition during cocaine-SA extinction reduces cocaine seeking

**Figure 3:**
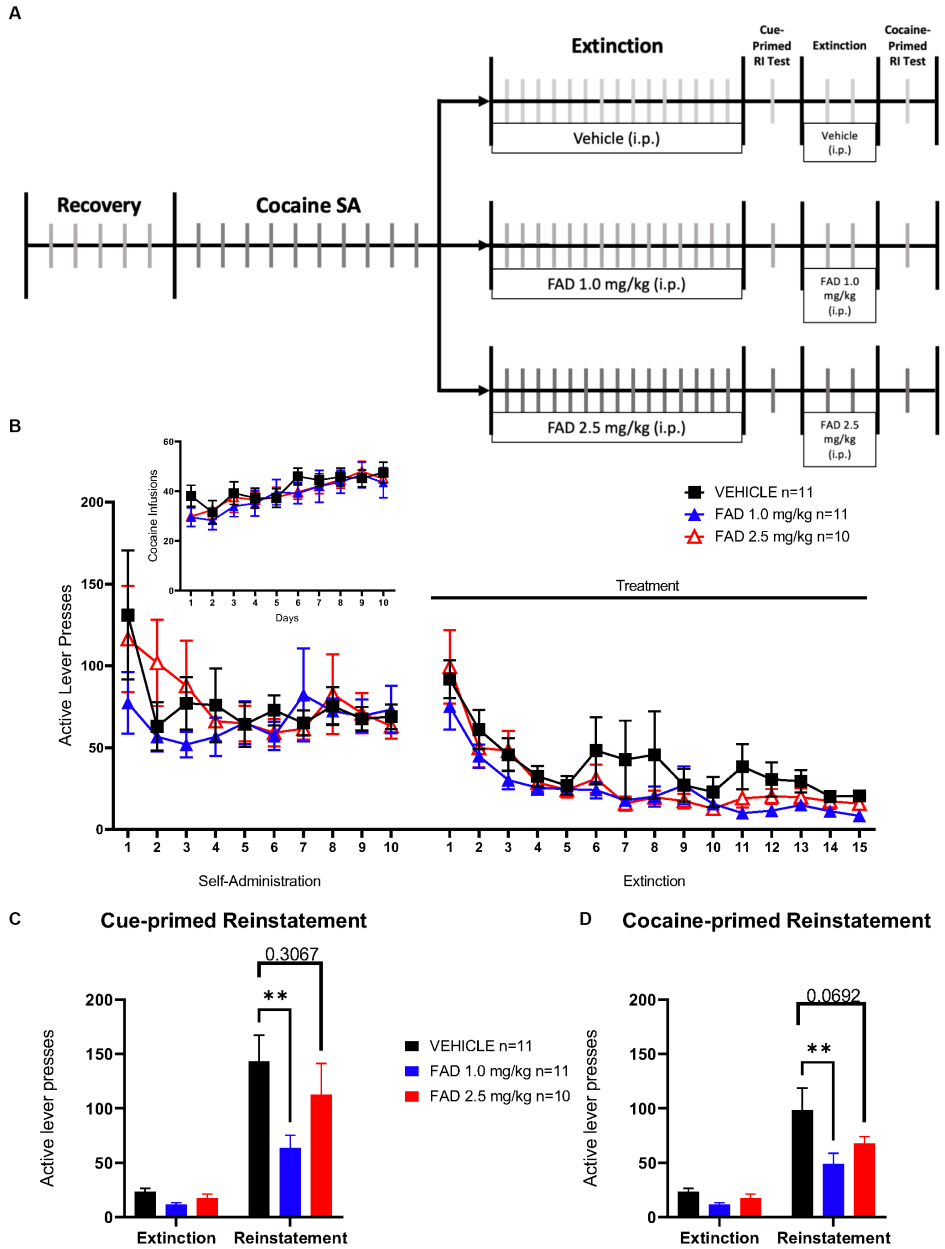
Reinstatement of drug seeking was significantly diminished by 1.0 mg/kg but not 2.5 mg/kg FAD. **(A)** Experimental timeline of cocaine SA showing i.p. injection of treatment (vehicle, FAD 1.0 mg/kg, FAD 2.5 mg/kg) during extinction, cue- and cocaine-primed reinstatement. **(B)** Active lever presses during SA and extinction sessions did not differ between treatments (vehicle, *n* = 11; FAD 1.0 mg/kg, *n* = 11; FAD 2.5 mg/kg, *n* = 10) across days. Insert shows number of cocaine infusions during SA. **(C)** Reinstatement to drug-paired cues was measured by active lever presses compared to average number of lever presses on the last three days of extinction for each group (left). Low dose FAD (1 mg/kg) significantly reduced cue-primed reinstatement as compared to vehicle. **(D)** Reinstatement to cocaine was measured by active lever presses as compared to average number of lever presses on the last three days of extinction for each group (left). Low dose FAD (1 mg/kg) significantly reduced cocaine-primed reinstatement as compared to vehicle. **p < 0.01.

Additionally, we examined the possibility that inhibition of aromatase activity during extinction of SA could affect extinction learning, and cue- and cocaine-induced seeking behavior in the male rats. As in the experiment described in Figure 3, rats were subdivided into three treatments (vehicle, FAD 1.0 mg/kg, and FAD 2.5 mg/kg; Figure 3A). Two Way ANOVA revealed no statistical difference in all groups for active lever pressing during conditioning and extinction phases (Figure 3B; *F*_48,696_ = 0.7647, *p* = 0.8769), or for cocaine infusions (Figure 3 Insert; *F*_18,261_ = 0.5605, *p = 0.9252*). On the other hand, all groups showed significant differences in active lever presses during cue-induced reinstatement compared with the average of the last three days of extinction (Figure 3C; *F*_1,29_ = 57.72, *p* < 0.0001). In addition, within cue-induced reinstatement groups, 1.0 mg/kg FAD reduced active lever presses compared to the vehicle group (*p* = 0.0013) while the 2.5 mg/kg FAD group showed similar active lever presses compared to the vehicle group (*p* = 0.3067). All groups showed a significant increase in cocaine-primed reinstatement (Figure 3D; *F*_1,29_ = 53.71, *p* < 0.0001) compared to the average of 2 days of extinction. Similar to cue-primed reinstatement, only the FAD 1.0 mg/kg group had a lower cocaine-primed reinstatement relative to the vehicle (*p* = 0.0016). In the 2.5 mg/kg FAD group cocaine-primed reinstatement was similar to the vehicle group (*p* = 0.0692).

### 3.4 Fadrozole did not affect non-drug reward

**Figure 4:**
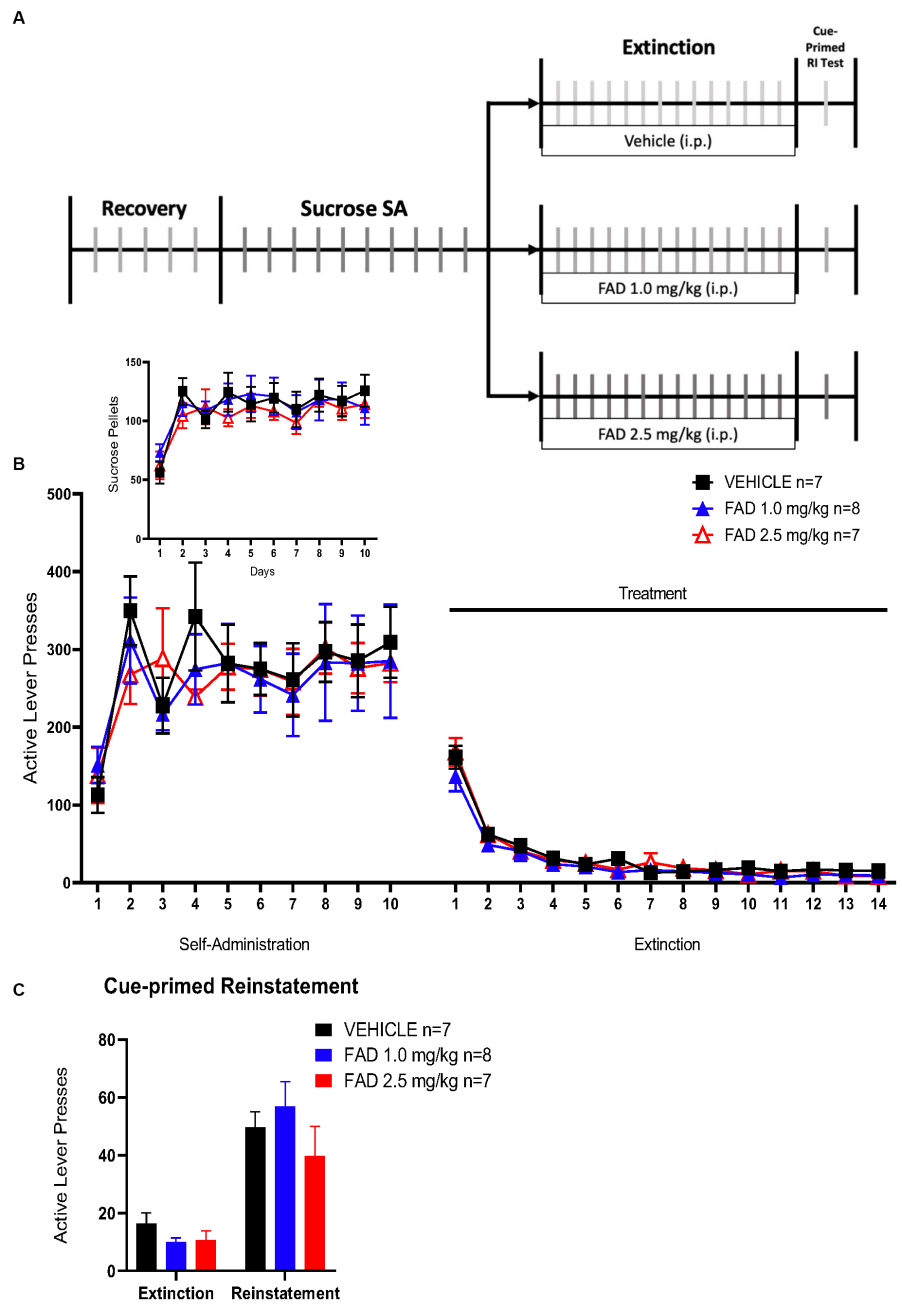
FAD had no effect on extinction and reinstatement of sucrose SA. **(A)** Schematic illustration of sucrose SA timeline including i.p. injection of treatment (vehicle, FAD 1.0 mg/kg, FAD 2.5 mg/kg) during extinction, and cue-primed reinstatement. **(B)** Active lever presses during SA and extinction sessions showed no significant differences between treatments across days and in number of sucrose pellets acquired in SA (Insert). **(C)** Reinstatement to sucrose was measured by active lever presses as compared to average number of lever presses on the last three days of extinction for each group (left). No effect of FAD was observed.

Based on results from cocaine SA experiments, we conducted a sucrose SA experiment to determine if FAD affects non-drug reward memory. This experiment had the same parameters as the cocaine SA (Figure 4A), except that the reward was sucrose pellets. There was no statistical difference, by a two way ANOVA, in active lever presses, sucrose pellets, extinction, or cue-primed reinstatement between all groups (Figure 4B, *F*_46,437_ = 0.4217, *p* = 0.9997; Figure 4 Insert, *F*_18,171_ = 0.6049, *p* = 0.8925; Figure 4C, *F*_2,19_ = 1.692, *p* = 0.2108). All groups did show cue-primed reinstatement of sucrose seeking when compared to the average of the last days of extinction (Figure 4C; *F*_1,19_ = 73.77, *p* < 0.0001).

## 4 Discussion

### 4.1 Fadrozole dose-dependently alters CPP extinction

The findings of this study demonstrate that FAD, an aromatase inhibitor that halts E2 synthesis, administered during extinction training dose-dependently alters extinction of cocaine CPP in male rats. A lower dose of FAD was observed to facilitate extinction, whereas a higher dose of FAD appeared to impede or prevent extinction. Results from Experiments 1 and 2 on CPP showed different outcomes on the first day of extinction. In Experiment 1, CPP expression was dampened, whereas in Experiment 2 it remained unaltered. This difference may be related to the more extended training in Experiment 2, relative to Experiment 1, as it leads to a stronger CPP memory that, in turn, may not be affected by lower doses of FAD during memory recall. These findings suggest that aromatase activity, which results in E_2_ synthesis, has a role in extinction learning of cocaine CPP in male rats. Consistent with our findings on high dose FAD (2.5 mg/kg), previous research has provided evidence supporting the role of estrogens in the modulation of extinction learning (Graham and Milad, 2013, 2014; Twining et al., 2013). Specifically, Twining et al. demonstrated that estrogen deprivation through ovariectomy in female rats impaired extinction learning, and resulted in perseverative cocaine seeking (Twining et al., 2013). Similarly, studies of fear extinction also implicate estrogens as necessary for extinction, demonstrating that lack of estrogens impairs the extinction memory formation in assigned female at birth (AFAB) persons and female rats (Graham and Milad, 2013). In 2014, Graham and Milad reported that male rats that received FAD had an impairment of extinction recall. Altogether, these results revealed a dose-dependent effect of the aromatase inhibitor, with higher concentrations leading to longer periods of cocaine seeking (more preference for the chamber paired with cocaine), than lower concentrations. In contrast, a lower dose appeared to facilitate extinction learning or blunt CPP expression. Our findings indicate that estradiol plays a vital role in extinction memory formation.

### 4.2 Fadrozole administration during SA extinction impairs cue- and cocaine-induced reinstatement

In order to determine whether FAD during extinction has an impact on cue- or cocaine-induced seeking behavior, we decided to perform extinction and reinstatement using cocaine SA. Results showed that aromatase inhibition during the extinction of cocaine SA had no effect on extinction learning. However, cue- and cocaine-primed reinstatement were significantly reduced by the lower dose of FAD (1 mg/kg), while the higher dose of FAD did not impair extinction nor cue- or cocaine-primed reinstatement. The mechanism by which a low dose of FAD alters cocaine-induced adaptations during extinction (withdrawal) within the reward circuitry remains to be determined. Our results show that inhibiting aromatase during extinction of cocaine SA does not affect extinction training. Nevertheless, a low dose of FAD may have an additive effect of increasing neurotransmitters during the 15-day treatment, thus creating a stronger memory of extinction and reducing cue and drug-induced reinstatement of cocaine seeking. FAD is known to reduce sexual behavior in male rats (Bonsall et al., 1992), and this could correlate with a more generalized reduction of motivational aspects (including cue and drug-primed SA reinstatement). Additionally, E2 is known to play a crucial role in synaptogenesis and memory formation by regulating transcriptional activity and protein synthesis (Torres, 2022). When E2 levels decrease, this can obstruct estrogen-induced memory formation by inhibiting transcription resulting in memory impairment. Consequently, this may cause a reduction in both cue- and drug-primed SA reinstatement. Interestingly, two different doses of FAD did not affect non-drug reinstatement when using a sucrose SA paradigm. This suggests that FAD does not alter the motivational properties of reward-associated behavior or memory formation. Clearly, further research is required to test these hypotheses, but taken as a whole, these findings suggest that a decrease in aromatase activity (i.e., less E2) has a direct impact on cocaine-seeking behavior in males.

### 4.3 Fad impacts the extinction learning process in the CPP paradigm but not in the SA paradigm

Both behavioral paradigms presented in this study have distinct features, allowing for assessment of different aspects of cocaine use disorder. Cocaine CPP is a form of Pavlovian conditioning, which links a context to reward (Bardo and Bevins, 2000; Tzschentke, 2007). CPP has operant-like components that influence behavior in drug-paired contexts (Green and Bardo, 2020) which can be taken as a measure of interoceptive reward without motivational aspects. On the other hand, SA involves operant conditioning through reinforcement (Panlilio and Goldberg, 2007) indicating that it measures interoceptive reward modulated by motivation.

Our results show that a high dose of FAD impairs extinction of CPP, but it has no effect on extinction in a SA paradigm. This suggests that FAD impairs extinction of a reward seeking (CPP) associated with the environment (cocaine-paired chamber), while having no effects on the motivational aspect of the operant conditioning paradigm (SA) during extinction. Further experiments are required to evaluate the disparity between the results from the CPP vs. SA paradigms.

### 4.4 Contrast: sex differences

The effect of E2 on reinforcing effects of cocaine in males remains unclear. Preclinical studies showed that administering E2 during SA had no effect on gonadectomized male rats (Jackson et al., 2006), while chronic administration of E2 increased cocaine intake in concurrent reinforcement and under progressive ratio (Bagley et al., 2019). It is important to note that these studies only examined the effect of E2 on the SA (reinforcing effects of cocaine), and not during extinction learning. In our study, we observed how FAD high dose, which disrupts E2 synthesis during cocaine extinction, impairs male cocaine-seeking behavior similar to what was previously seen in females (Twining et al., 2013). On the other hand, low dose FAD facilitated extinction or suppressed CPP expression in male rats, suggesting sex differences in E2mediation of cocaine seeking. Our findings indicate that E2 is important in both sexes for cocaine seeking and extinction.

### 4.5 Possible mechanism

The dose–response effects observed during CPP extinction and SA cue- and cocaine-induced reinstatement might be due to the effects of FAD on other molecules from the estrogen synthesis pathway (for example, testosterone, or last metabolites in this pathway before being aromatized to estrone and E2 by CYP19), as a compensatory mechanism, or on some other receptor. We speculate that upon decreasing aromatized activity with FAD, testosterone levels increase due to not being converted to E2 (Soma et al., 2000; Cardone et al., 2002). The increased testosterone levels can activate another pathway through the five ⍺ or β reductase enzymes, producing 5-Dihydrotestosterone (5-DHT). A study with FAD administration into the zebrafish brain showed that not only was E2 decreased, but 5β reductase (5 DHT precursor enzyme) activity was increased (Wade et al., 1994). These studies suggest that testosterone metabolites can contribute to changes in the brain, possibly mediating cocaine extinction learning and memory. Another recent study showed that increased testosterone levels by acute administration of a gonadotropin-releasing hormone (GnRH) receptor agonist enhanced extinction recall during fear extinction training in rats (Maeng et al., 2017). Clearly, additional studies are needed to elucidate if and how testosterone metabolites modulate cocaine-seeking behavior.

Alternatively, estrogen receptor activation (ER⍺, ERβ) by 17β-estradiol leads to many critical physiological processes ranging from neuroprotection to effects on learning and memory (McEwen and Alves, 1999; Dumitriu et al., 2010). One possibility is that a decrease in E2 availability reduces interaction with its receptors, and reduced receptor activation could explain part of the duality observed when FAD was administered, causing impairment in extinction behavior. E2 scarcity, due to a higher inhibition of aromatase inhibitor, would result in no activation of estrogen receptors. On the contrary, a lower dosage of aromatase inhibitor might not fully affect E2 levels entirely. Instead, it may partially halt E2 synthesis, with a low level of E2 present that could trigger a compensatory mechanism within a cell, and generate more receptors, thus facilitating extinction and lowering reinstatement. Low concentrations of both estrogens and androgens have been shown to upregulate receptor production (Smith et al., 2002). Moreover, there is new evidence showing sex differences in expression of E2 and androgen receptors within the projections from the medial preoptic area to the ventral tegmental area in rats (Martz et al., 2023). This difference in hormone receptor expression between sexes may contribute to the behavioral effects of E2 on reward and motivation pathways in males and females. Further studies should examine the pharmacodynamics of FAD at both doses, and measure estrogen receptor activity in brain structures related to reward.

Estrogens have an impact on various neurotransmitter systems and can upregulate and increase their activity. These systems include dopamine (DA), glutamate, and serotonin (5HT), with estrogens modulating their synthesis, receptors, and transporters. These neurotransmitters are essential for learning and memory processes (Becker, 1990, 2009; Sumner and Fink, 1995; Gazzaley et al., 1996; Pecins-Thompson et al., 1996; Ramirez and Zheng, 1996; Bethea et al., 2000; Osterlund et al., 2000; Adams et al., 2004; Maharjan et al., 2005; Oberlander and Woolley, 2016; Krolick et al., 2018). Our data suggest that a low dose of FAD slightly increases estrogen levels, which in turn may improve the release of neurotransmitters and result in facilitated CPP extinction learning. On the other hand, a high dose of FAD could cause a more significant decrease in estrogen levels, which would have the opposite effect.

Additionally, some drugs have a biphasic dose–response curve, which means that low and high doses have opposite effects. This could be due, for example, to different receptor affinities, or to differential modulation of downstream signaling pathways at different concentrations. Further research is necessary to assess these and other related hypotheses.

### 4.6 Study limitations

Since the behavioral effects observed in this study are due to intraperitoneal FAD injections, the question may arise whether FAD can cross the brain–blood barrier. Wade et al. (1994) showed that FAD reduced aromatase activity in the brain demonstrating that it can cross the brain–blood barrier. Although we did not collect blood samples or brain tissue from experimental rats, multiple preclinical studies showed that aromatase inhibitors, such as FAD, reduced levels of estrogens in the blood (Soma et al., 2000; Cardone et al., 2002) as well as aromatase activity (Ankley et al., 2002; Villeneuve et al., 2006).

Another limitation of this study is that we focused on the effect of FAD during cocaine extinction within both paradigms but only on SA reinstatement. To learn more about this caveat and determine whether or not it has any bearing on the observed SA data, future experiments could look into the possibility of changes in CPP reinstatement and sucrose-induced CPP.

### 4.7 Closing remark

The observed results are consistent with previous research that has highlighted the significance of estrogens in the developmental processes of the male brain (Wu et al., 2009) as well as their role in providing neuroprotection (Zendedel et al., 2018; Wang et al., 2021). Although there is no FDA-approved pharmacologic agent for cocaine use disorder, sex-difference studies have highlighted hormones, mainly estrogens, as essential elements influencing this disorder. This study adds new information on how sex steroid hormones (like E2) directly or indirectly regulate drug seeking. Our findings suggest that E2 modulates cocaine extinction learning and cocaine-seeking behavior in male rats. Altogether, these results, along with all updated knowledge about estrogens as a whole, will prompt a reassessment of the significant impact estrogens have on sex differences in cocaine use disorder, and how estrogens can facilitate the recovery of those affected by this disorder. Discovering a new mechanism to diminish cocaine relapse will help many women and men overcome cocaine use disorder, ultimately improving their lifestyle.

## Data availability statement

The raw data supporting the conclusions of this article will be made available by the authors, without undue reservation.

## Ethics statement

The animal study was approved by Ponce Health Sciences University Institutional Animal Care and Use Committee. The study was conducted in accordance with the local legislation and institutional requirements.

## Author contributions

JA-T: Conceptualization, Formal analysis, Investigation, Methodology, Resources, Supervision, Validation, Writing – original draft, Writing – review & editing. RM-S: Investigation, Writing – original draft, Writing – review & editing. AS: Formal analysis, Investigation, Writing – original draft. GR-T: Formal analysis, Investigation, Validation, Writing – original draft. JP-T: Formal analysis, Investigation, Writing – review & editing. YP-P: Investigation, Writing – original draft, Writing – review & editing. DM: Investigation, Methodology, Resources, Supervision, Writing – review & editing. MS-O: Conceptualization, Formal analysis, Investigation, Methodology, Resources, Supervision, Validation, Writing – original draft, Writing – review & editing.
